# Disturbed Expression of EphB4, but Not EphrinB2, Inhibited Bone Regeneration in an In Vivo Inflammatory Microenvironment

**DOI:** 10.1155/2016/6430407

**Published:** 2016-12-18

**Authors:** Li-Li Shen, Li-Xia Zhang, Li-Mei Wang, Rong-Jing Zhou, Cheng-Zhe Yang, Jin Zhang, Pi-Shan Yang

**Affiliations:** ^1^Shandong Provincial Key Laboratory of Oral Tissue Regeneration, Shandong University, Jinan, Shandong, China; ^2^Liaocheng People's Hospital, Liaocheng, Shandong, China; ^3^Jinan Stomatological Hospital, Jinan, Shandong, China; ^4^Department of Periodontology, School of Dentistry, Shandong University, Jinan, Shandong, China; ^5^Department of Oral & Maxillofacial Surgery, Qilu Hospital and Institute of Stomatology, Shandong University, Jinan, Shandong, China; ^6^Department of Endodontology, School of Dentistry, Shandong University, Jinan, Shandong, China

## Abstract

The important role of ephrinB2-EphB4 signaling pathway in bone remodeling has been well established. However, it is still unclear whether this bidirectional signaling also has effects on the regenerative processes of bone defects created in an inflammatory microenvironment. In this study, an experimental animal model of bone defects treated with lentiviruses was prepared and an inflammatory microenvironment was established. Expression levels of bone marker genes were monitored in the newly formed bone tissue using quantitative reverse transcriptase polymerase chain reaction and western blot. Immunohistochemical (IHC) staining and histomorphometric analysis were also performed to evaluate bone healing processes. Compared with the pLenti6.3-ctrl group, the pLenti6.3-ephb4siRNA group exhibited lower expression levels of bone formation marker genes and a higher level of NFATc1 in the new bone tissue. In addition, the newly formed bone was thinner and the number of giant osteoclasts was higher in the pLenti6.3-ephb4siRNA group than that in the pLenti6.3-ctrl group. In contrast, there was no significant difference between the pLenti6.3-efnb2siRNA group and the pLenti6.3-ctrl group. In conclusion, EphB4 plays an irreplaceable role in bone regeneration in an inflammatory microenvironment, whereas the functional loss of ephrinB2 can be effectively compensated, most possibly by other ephrins with similar chemical structures.

## 1. Introduction

Continuously occurring on existing bone surfaces, bone remodeling starts from old bone resorption by osteoclasts, followed by new bone regeneration in the resorption lacunae by osteoblasts [1]. It is essential to preserve a certain balance between bone resorption and bone formation to maintain the structural integrity and mechanical function of the skeleton throughout life and especially during bone repair after injury [2, 3]. To that end, a complex and delicate communication should be established between osteoblasts and osteoclasts [4]. This tightly coupled communication is prerequisite to ensuring that microscopic skeletal damage is repaired and the aged bone replaced to maintain a functional skeletal system [5].

During the process of bone remodeling, macrophage-colony stimulating factor (M-CSF) and receptor activator of nuclear factor kappa-B ligand (RANKL) produced by osteoblasts stimulate their receptors on osteoclast precursors, activate downstream molecular pathways, and promote the differentiation of osteoclasts [6]. On the other hand, tartrate-resistant acid phosphatase (TRAP) released by osteoclasts has been reported to stimulate bone formation [7]. In 2006, Zhao and colleagues reported that Eph-ephrin bidirectional signaling mediates interaction between osteoclast and osteoblast populations [8]. Ephs are a large family of tyrosine kinase receptors which can be divided into EphAs and EphBs based on gene sequence and binding affinity to the ephrin ligands [9]. Acting as contact-dependent repellent molecules in both prenatal and postnatal organisms, Ephs play various roles in boundary formation, axon guidance, angiogenesis, bone formation, and bone homeostasis [10, 11]. Briefly in maintaining homeostasis of bone remodeling, osteoclasts express ephrinB2, a transmembrane ligand, while osteoblasts express the EphB4 receptor. The reverse signaling through ephrinB2 into osteoclast precursors suppresses osteoclast differentiation and the forward signaling through EphB4 into osteoblasts enhances osteogenic differentiation. In addition, in vivo overexpression of EphB4 in osteoblasts increases bone mass by enhancing bone formation and decreasing bone resorption in a mouse model [8]. Furthermore, EphB4 was reported to be important in osteogenic differentiation and migration of human bone marrow-derived mesenchymal stem cells (hBM-MSCs) [12], and ephrinB2-EphB4 signaling successfully promotes osteoblastic mineralization in vitro [13].

As discussed above, the ephrinB2-EphB4 bidirectional signaling system shows a significant regulatory effect on bone formation and bone remodeling under physiological conditions, which is a potential target for the development of novel therapeutics to enhance bone regeneration. However, a high percentage of patients visiting dental clinics suffer from local chronic inflammation such as periodontitis, peri-implantitis, and periapical periodontitis and it is not clear whether EphB4 receptor and ephrinB2 ligand exerted similar effect on bone defect regeneration in inflammatory and noninflammatory microenvironment. In this study, in vivo expression of ephrinB2 or EphB4 in mandibular bone defects created in an inflammatory mouse model was knocked down using siRNAs specifically targeting ephrinB2 or EphB4, respectively. Bone formation and bone resorption in the bone defects were evaluated to fully elucidate the function of ephrinB2-EphB4 bidirectional signaling in an inflammatory microenvironment.

## 2. Materials and Methods

### 2.1. Synthesis of Small Interfering RNAs (siRNAs) Specifically Targeting EphrinB2 or EphB4

siRNAs specifically targeting ephrinB2 or EphB4 were designed and synthesized by ThermoFisher Scientific, Inc. (Shanghai, China). The negative control siRNA with no homology to any known mouse or human gene was also synthesized to serve as a negative control. Synthesized siRNAs were duplexed and ligated into the pcDNA6.2-GW/EmGFP-miR using the BLOCK-iT Pol II miR RNAi Expression Vector Kit with EmGFP (ThermoFisher Scientific, Shanghai, China). According to the published mRNA sequences, mouse* Efnb2* (gene access number: NM_010111.5) and* Ephb4* (gene access number: NM_001159571.1) genes were synthesized using DNA synthesis technology and overlapping PCR by ThermoFisher Scientific, Inc. (Shanghai, China). The genes were then sequenced and subcloned into pcDNA3.1(+) (ThermoFisher Scientific, Shanghai, China) between restriction sites HindIII and BamHI.

To validate siRNA-mediated knockdown of ephrinB2 or EphB4 mRNA, pcDNA6.2-GW/EmGFP-miR plasmids containing the siRNA inserts were cotransfected along with pcDNA3.1(+)-ephrinB2 or pcDNA3.1(+)-EphB4 into HEK-293 human embryonic kidney cells (American Type Culture Collection (ATCC), Manassas, VA) using Lipofectamine 2000 Reagent (ThermoFisher Scientific, Shanghai, China) according to the manufacturer's instructions. HEK-293 cells were chosen for validating siRNA-mediated knockdown of targeted mRNAs for their high transfection efficiency. Forty-eight hours after transfection, cells were trypsinized and the total RNA was isolated using the TRIzol reagent (ThermoFisher Scientific, Shanghai, China). The first strand of cDNA was generated using SuperScript III reverse transcriptase (ThermoFisher Scientific, Shanghai, China). A quantitative real-time reverse transcription-PCR (qRT-PCR) assay was performed by ThermoFisher Scientific, Inc. (Shanghai, China) to detect the mRNA levels of mouse ephrinB2 or EphB4. Relative differences in the PCR product amounts were evaluated by the comparative cycle threshold (CT) method, with Glyceraldehyde-3-Phosphate Dehydrogenase (GAPDH) serving as a control. The sequences of the primers used in siRNA validation experiment were 5′-ATCAGCCAGGAATCACGGTC-3′ and 5′-TGTGGAGAGTGTTTGCGGTGTC-3′ for ephrinB2; 5′-AGCCTCACTATTCTGCTTTCGG-3′ and 5′-GATTTTCTTCTGGTGTCCTGCC-3′ for EphB4; and 5′-GAAGGTCGGAGTCAACGGATT-3′ and 5′-CGCTCCTGGAAGATGGTGAT-3′ for GAPDH.

### 2.2. Preparation of Lentiviral Particles Expressing siRNAs Targeting Mouse EphrinB2 or EphB4

After siRNA validation, selected siRNAs specifically targeting mouse ephrinB2 or EphB4 were introduced into the pDONR221 vector by in vitro recombination with the BP-clonase II enzyme mix (ThermoFisher Scientific, Shanghai, China). The resulting BP-recombination plasmids were then transferred into the lentiviral expression vector pLenti6.3/V5-DEST by in vitro recombination with the LR-clonase II enzyme mix (ThermoFisher Scientific, Shanghai, China). The resulting lentiviral vectors were named as pLenti6.3-efnb2siRNA and pLenti6.3-ephb4siRNA, respectively. A lentiviral vector encoding the above-mentioned negative control siRNA, pLenti6.3-ctrl, was also created to be used as a negative control.

The pLenti6.3-efnb2siRNA, pLenti6.3-ephb4siRNA, or pLenti6.3-ctrl lentiviral vector was transfected into human 293T cells with ViraPower packing mix using Lipofectamine 2000 (ThermoFisher Scientific, Shanghai, China). Forty-eight hours after the transfection, the supernatant was collected, centrifuged at 3,000 rpm for 10 min to remove cell debris, and filtered with 0.45 *µ*m filters. The lentiviral particles were then concentrated by centrifugation at 50,000*g* for 2 hours, resuspended in opti-MEM, and subjected to titration. After the titration, the lentiviral particles were diluted to the concentration of 1 × 10^8^ transducing units per milliliter (TU/mL) and stored at −80°C.

### 2.3. Establishment of an In Vivo Inflammatory Microenvironment* via* Intraperitoneal Injections of TNF-*α* into Mice

**Figure 1:**
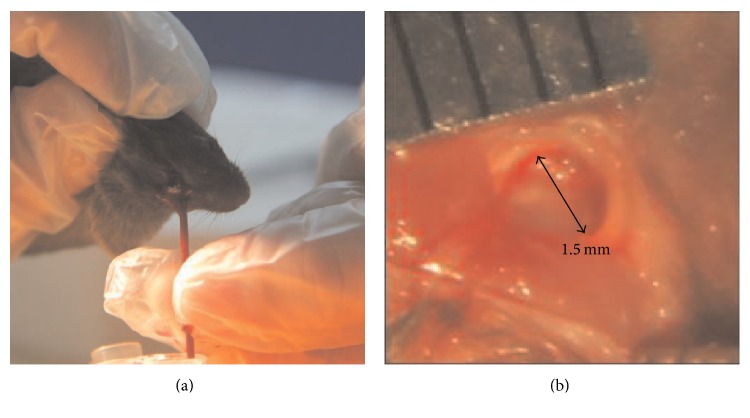
Animal surgery. (a) Blood samples were collected from the angular vein of the mice. (b) Bone defects, 1.5 mm in diameter, were created on the alveolar bone overlying the mesial root of the right lower first molar of the mice.

Twenty-four 6–8-week-old male C57BL/6 mice were randomly assigned into 4 groups to receive intraperitoneal injections of TNF-*α* at the doses of 0 *µ*g/kg, 0.5 *µ*g/kg, 3 *µ*g/kg, or 5 *µ*g/kg, respectively. The injections were performed every other day. At 3, 7, 10, and 14 days after the first injection, blood samples were collected from the angular vein of the animals (Figure 1(a)), and the serum concentration of TNF-*α* was determined using the mouse TNF-alpha Platinum ELISA kit (eBioscience, San Diego, CA, USA) according to the protocols provided by the manufacturers.

All animals used in this study were maintained and used in accordance with guidelines established by the Institutional Animal Care and Use Committee of Shandong University in Jinan, Shandong Province, China.

### 2.4. Animal Surgery

After being anesthetized* via* intraperitoneal injections of a mixture of Ketamine (80 mg/kg) and Xylazine (10 mg/kg), the alveolar bone overlying the mesial root of the right lower first molar of male C57BL/6 mice was exposed. Bone defects, 1.5 mm in diameter, were then created in this region [14] using a dental bur under continuous irrigation with phosphate-buffered saline (PBS) (Figure 1(b)). Randomly assigned into three groups, the bone defects were treated with the pLenti6.3-efnb2siRNA, pLenti6.3-ephb4siRNA, or pLenti6.3-ctrl lentiviral particles, respectively, and each bone defect was injected with 5 *μ*L of the lentiviral particles using a microsyringe (Asone, Japan). The tissue was then closed with 4-0 gut suture. From the day of surgery, all animals received intraperitoneal injections of TNF-*α* at the dose of 5 *µ*g/kg, and the injections were performed every other day. At days 7, 14, and 21 after surgery, the mice were sacrificed and the mandibles were isolated for further analysis.

### 2.5. Real-Time RT-PCR Analysis

The overlying soft tissues were carefully removed, and the bone tissues bordering the defect area (about 1.5 × 1.5 mm) were dissected from the mandible, snap-frozen in liquid nitrogen. Total RNA was extracted from the bone tissues with TRIzol reagent (ThermoFisher Scientific, Carlsbad, CA, USA) and reverse-transcribed using the PrimerScript RT Reagent Kit with gDNA Eraser (TaKaRa, Shijodori Kyoto, Japan). Real-time PCR was performed using the Light Cycler Fast Start DNA Master SYBR Green I (Roche Applied Science, Penzberg, Germany). The sequences of the primers for amplification of mouse Runx2, OC, Osx, BSP, ALP, NFATc1, ephrinB2, EphB4, and GAPDH were listed in Table 1. mRNA expression was normalized to the GAPDH housekeeping gene by the 2^−ΔΔCT^ method. Each experiment was evaluated with three PCR reactions and each experiment was repeated for three times.

### 2.6. Western Blot Analysis

Total protein was isolated and determined using a BCA protein assay kit (Beyotime, Beijing, China). Western blot analysis was then performed using NuPAGE 4–12% Bis-Tris gradient gels and 0.45 *µ*m Invitrolon polyvinylidene fluoride membranes (ThermoFisher Scientific, Carlsbad, CA, USA). Antibodies for Runx2 (1 : 1000, Abcam, Cambridge, MA), BSP (1 : 500, Santa Cruz Biotechnology, Santa Cruz, CA), and *β*-actin (1 : 500, Santa Cruz Biotechnology, Santa Cruz, CA) were used. The secondary antibodies were horseradish peroxidase- (HRP-) linked goat-anti-rabbit IgG (Santa Cruz Biotechnology, Santa Cruz, CA). Blots were visualized using ECL chemiluminescence reagents from Pierce Biotechnology (Rockford, IL, USA). Images of blots were analyzed using Image 4.11 (Dahui Biotechnology, Guangzhou, China).

### 2.7. Histomorphometric Analysis

The mandibular bone was fixed with 4% paraformaldehyde (PFA, Sigma-Aldrich, Saint Louis, MO), decalcified in 10% EDTA, and embedded in paraffin. Tissue sections, 5 *µ*m in thickness, were cut in the coronal direction and numbered. The tissue sections positioned in the central region of the bone defects were collected for quantitative analysis and stained with hematoxylin and eosin (HE). Images were taken with an Olympus microscope (Olympus, Japan) and the ProgRes CapturePro software (Jenoptik Optical Systems, Germany). The newly formed bone area was expressed as a percentage (area of newly formed bone/area of original wound) and was measured with the Image-Pro Plus 6.0 software (Media Cybernetics, Silver Spring, MD, USA) using a double-blinded method to prevent observer's bias.

### 2.8. Immunohistochemical Staining (IHC Staining)

Primary antibodies against Runx2 (1 : 1000) and OC (1 : 500) were purchased from Abcam (Cambridge, MA, USA). Control sections were incubated with normal rabbit serum without the primary antibodies. After rinsing with the running water for 5 min, sections were counterstained with hematoxylin for 30 sec. Images were taken with an Olympus microscope (Olympus, Japan), and integrated optical densities (IOD) of Runx2 and OC in all of the three groups were measured using the Image-Pro Plus 6.0 software (Media Cybernetics, Silver Spring, MD, USA). For each sample, three different sections were selected at the central part of the bone defect for IOD measurement. For each section, IOD was measured in 4 random fields with the same area (40x magnification) in the bone defect region.

### 2.9. Tartrate-Resistant Acid Phosphatase (TRACP) Staining

TRACP staining was performed using the Acid Phosphatase, Leukocyte (TRAP) Kit purchased from Sigma-Aldrich (Saint Louis, MO). Briefly, slides were incubated at 37°C for 30 minutes in AS-BI phosphate (0.4 mg/mL) in acetate-tartrate buffer (200 mM sodium acetate, 100 mM potassium sodium tartrate, pH 5.2). The slides were then transferred into sodium nitrite solution (1 : 1 v/v) in prewarmed tartrate-acetate buffer and incubated for 30 to 60 minutes at 37°C until osteoclasts were bright red. Sections were then counterstained with methyl green (Sigma-Aldrich, St. Louis, MO). Stained sections were observed using an Olympus microscope (Olympus, Japan) and TRACP-positive cells with three or more nuclei were counted.

### 2.10. Statistical Analysis

Statistical analysis was performed using the SPSS 19.0 Statistics Software (SPSS Inc., Chicago, IL, USA). All data were expressed as the mean ± standard error of the mean (SEM). Statistical significance was tested by Independent-Samples *t*-test. A* p* value below 0.05 was considered statistically significant.

## 3. Results

### 3.1. Lentiviral Particles Encoding siRNAs Targeting Mouse EphrinB2 or EphB4 Were Successfully Prepared and Titrated

**Figure 2:**
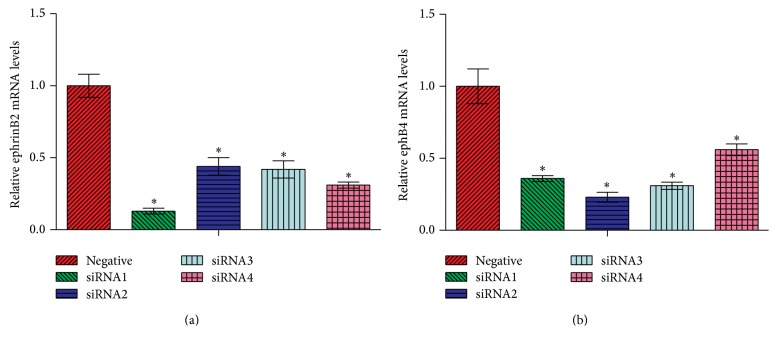
Evaluation of the efficiency of siRNAs in downregulating expression levels of the corresponding target genes. (a) Efficiency of siRNAs specifically targeting ephrinB2 in downregulating mRNA levels of ephrinB2. (b) Efficiency of siRNAs specifically targeting EphB4 in downregulating mRNA levels of EphB4. ^*∗*^ *p* < 0.05 versus negative control.

Four siRNAs each, specifically targeting the mouse ephrinB2 and EphB4 genes, were designed, synthesized, and ligated into the pcDNA6.2-GW/EmGFP-miR vector. To evaluate the efficiency of siRNAs in downregulating expression levels of the target genes, pcDNA6.2-GW/EmGFP-miR plasmids containing the siRNA inserts were cotransfected along with pcDNA3.1(+)-ephrinB2 or pcDNA3.1(+)-EphB4 into HEK-293 cells. Among the four siRNAs targeting ephrinB2, siRNA #1 demonstrated the highest inhibition efficiency, which successfully downregulated the mRNA level of ephrinB2 by 87% (Figure 2(a)). Similarly, among siRNAs targeting EphB4, siRNA #2 showed the highest inhibition efficiency, with the mRNA level of EphB4 downregulated by 77% (Figure 2(b)). Therefore, siRNA #1 targeting ephrinB2 and siRNA #2 targeting EphB4 were chosen for the lentivirus preparation experiment and were used in the following in vivo studies.

After a series of in vitro recombination assays, selected siRNAs were successfully transferred into the lentiviral expression vector pLenti6.3/V5-DEST and were named as pLenti6.3-efnb2siRNA and pLenti6.3-ephb4siRNA, respectively. pLenti6.3-efnb2siRNA, pLenti6.3-ephb4siRNA, and the negative control pLenti6.3-ctrl were then pseudotyped with the pLP1, pLP2, and pLP/VSVG envelope glycoproteins, respectively. After filtration, centrifugation, and titration, the lentiviral particles were diluted to 1 × 10^8^ transducing units per milliliter (TU/mL) with opti-MEM and stored at −80°C for further use.

### 3.2. An In Vivo Inflammatory Microenvironment Was Established in Mice* via* Intraperitoneal Injections of TNF-*α* at the Dose of 5 *µ*g/kg

**Figure 3:**
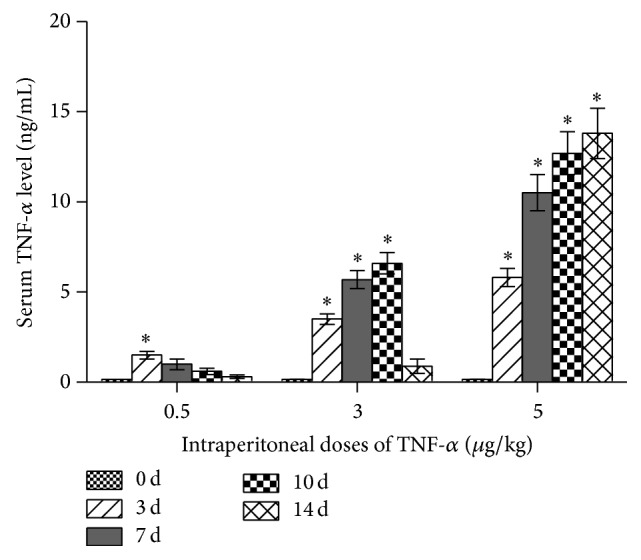
Serum TNF-*α* levels in male C57BL/6 mice receiving intraperitoneal injections of TNF-*α* at the doses of 0 *µ*g/kg, 0.5 *µ*g/kg, 3 *µ*g/kg, and 5 *µ*g/kg every other day for 3, 7, 10, and 14 days. Three independent experiments were performed in triplicate, and data were represented as mean ± SEM. ^*∗*^ *p* < 0.05, versus 0 d group.

After being injected with TNF-*α* intraperitoneally, blood samples were collected from the C57BL/6 mice and subjected for the determination of serum TNF-*α* level. We found that animals receiving injections of TNF-*α* at the dose of 0.5 *µ*g/kg only showed a slight increase in the serum TNF-*α* level. In mice receiving injections of TNF-*α* at the dose of 3 *µ*g/kg, serum TNF-*α* levels were continuously increased at 3 days, 7 days, and 10 days after the first injection. However at 14 days after the first injection, the increase in serum TNF-*α* level was less prominent. In contrast, in animals receiving injections of TNF-*α* at the dose of 5 *µ*g/kg, serum TNF-*α* level was dramatically increased from 3 days after the first injection and reached a steady plateau state thereafter (Figure 3). Therefore intraperitoneal injections of TNF-*α* at the dose of 5 *µ*g/kg were used to establish an inflammatory microenvironment in the following in vivo experiments.

### 3.3. Disturbed Expression of EphB4, but Not EphrinB2, Significantly Downregulated Expression Levels of Bone-Related Genes and Upregulated Expression Level of NFATc1 in an Inflammatory Microenvironment Created in Mice

**Figure 4:**
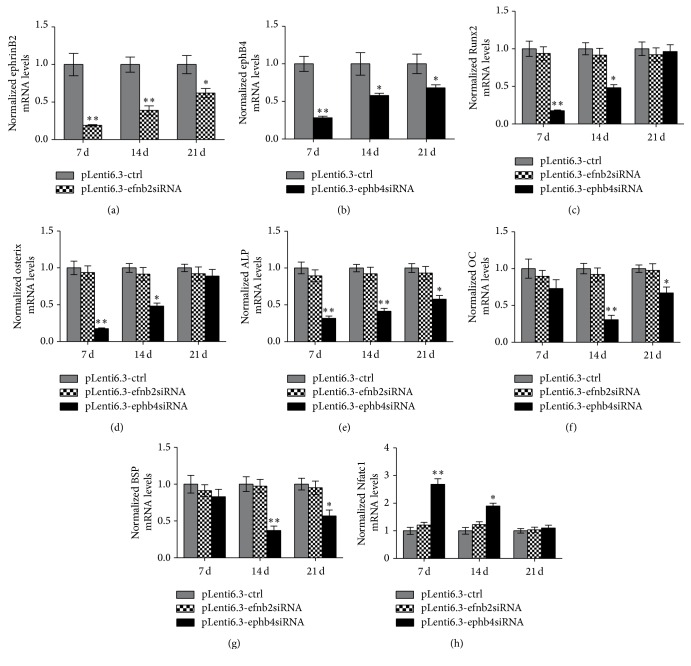
Disturbed expression of EphB4, but not ephrinB2, significantly downregulated mRNA levels of bone-related genes and upregulated the mRNA level of NFATc1 in an inflammatory microenvironment created in mice. (a) EphrinB2 mRNA levels were dramatically decreased in the pLenti6.3-efnb2siRNA group when compared with those in the pLenti6.3-ctrl group. (b) EphB4 mRNA levels were significantly downregulated in the pLenti6.3-ephb4siRNA group when compared with those in the pLenti6.3-ctrl group. (c–h) mRNA levels of bone-related genes such as Runx2 (c), Osx (d), ALP (e), OC (f), and BSP (g), as well as the osteoclastogenic transcription factor NFATc1 (h), were also monitored in the isolated newly formed bone tissue. Three independent experiments were performed in triplicate, and data were represented as mean ± SEM. ^*∗*^ *p* < 0.05, ^*∗∗*^ *p* < 0.001 versus pLenti6.3-ctrl group.

Mandibular bone defects were created in male C57BL/6 mice receiving intraperitoneal injections of TNF-*α* at the dose of 5 *µ*g/kg every other day. The bone defects were treated with pLenti6.3-efnb2siRNA, pLenti6.3-ephb4siRNA, or pLenti6.3-ctrl lentiviral particles, respectively. Mice were sacrificed at 7, 14, and 21 days after surgery, and the newly formed bone was isolated. We found that ephrinB2 mRNA levels in the newly formed bone tissue were dramatically decreased in the pLenti6.3-efnb2siRNA group, while the EphB4 mRNA levels were significantly downregulated by the treatment of pLenti6.3-ephb4siRNA lentiviral particles, when compared with the mRNA levels of the corresponding genes in the pLenti6.3-ctrl group (Figures 4(a) and 4(b)). In contrast, siRNAs specifically targeting ephrinB2 do not target EphB4, and vice versa, based on the nucleotide sequences. Furthermore, no statistically significant difference was detected in EphB4 expression levels between the pLenti6.3-efnb2siRNA group and the pLenti6.3-ctrl group and in ephrinB2 expression levels between the pLenti6.3-ephb4siRNA group and the pLenti6.3-ctrl group (data not shown).

In order to investigate the effect of the ephrinB2-EphB4 bidirectional signaling pathway on bone formation and bone resorption in an in vivo inflammatory microenvironment, expression levels of bone-related genes such as Runx2, Osx, ALP, OC, and BSP, as well as the osteoclastogenic transcription factor NFATc1, were also monitored in the isolated newly formed bone tissue. We found that mRNA levels of these above-mentioned bone-related genes were significantly lower in the pLenti6.3-ephb4siRNA group than in the pLenti6.3-ctrl group. In contrast, no significant difference in expression levels of bone-related genes was detected between the pLenti6.3-efnb2siRNA group and the pLenti6.3-ctrl group (Figures 4(c)–4(g)). Furthermore, NFATc1 mRNA levels were significantly increased in the pLenti6.3-ephb4siRNA group when compared with those in the pLenti6.3-ctrl group at 7 and 14 days after surgery. However, no significant difference was detected in NFATc1 mRNA levels between the pLenti6.3-efnb2siRNA group and the pLenti6.3-ctrl group (Figure 4(h)).

**Figure 5:**
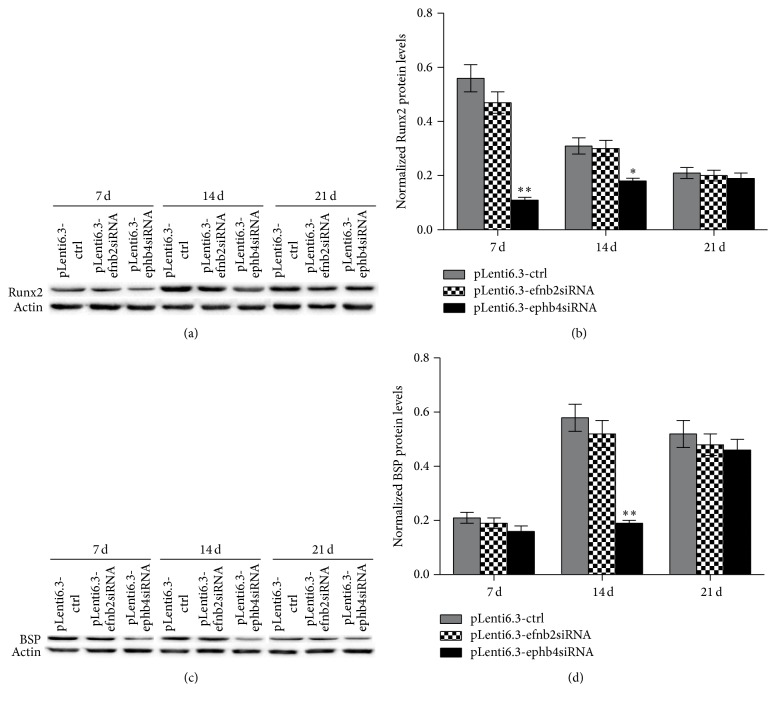
Disturbed expression of EphB4, but not ephrinB2, significantly downregulated protein levels of Runx2 (a-b) and BSP (c-d) in the newly formed bone tissue. Three independent experiments were performed in triplicate, and data were represented as mean ± SEM. ^*∗*^ *p* < 0.05, ^*∗∗*^ *p* < 0.001 versus pLenti6.3-ctrl group.

We also performed western blot analysis to determine the protein levels of Runx2 and BSP in the newly formed bone tissue. As shown in Figures 5(a) and 5(b), the protein levels of Runx2 were significantly lower in the pLenti6.3-ephb4siRNA group when compared with those in the pLenti6.3-ctrl group at 7 and 14 days after surgery. Similarly, the protein level of BSP was decreased in the pLenti6.3-ephb4siRNA group when compared with that in the pLenti6.3-ctrl group at 14 days after surgery (Figures 5(c) and 5(d)). Interestingly, no statistically significant difference in the protein levels of Runx2 and BSP was detected between the pLenti6.3-efnb2siRNA group and the pLenti6.3-ctrl group (Figures 5(a)–5(d)).

### 3.4. Disturbed Expression of EphB4, but Not EphrinB2, Significantly Retarded Bone Regeneration in an Inflammatory Microenvironment Created in Mice

**Figure 6:**
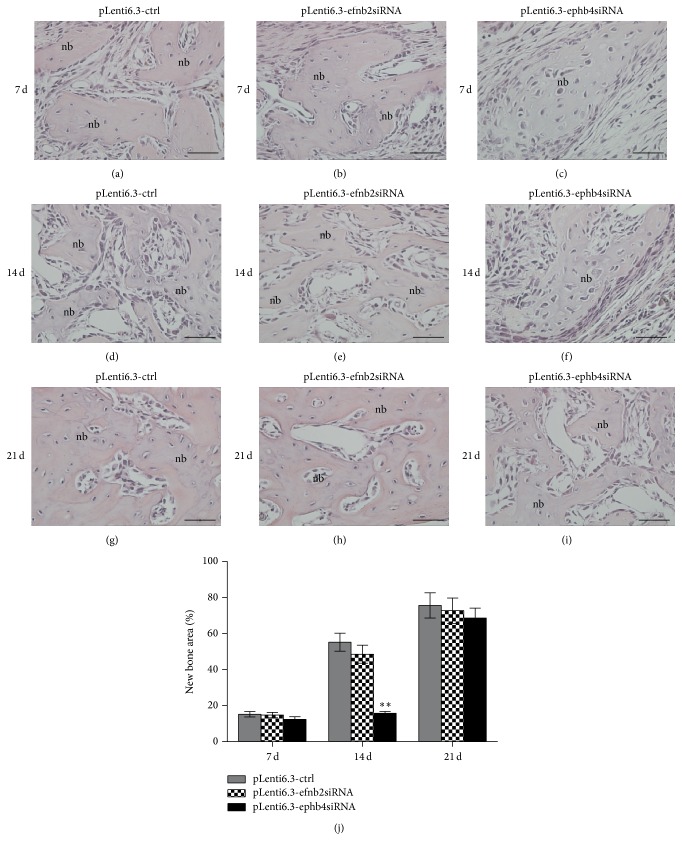
Disturbed expression of EphB4, but not ephrinB2, significantly retarded bone regeneration in an inflammatory microenvironment created in mice. (a–i) At 7 days (a–c), 14 days (d–f), and 21 days (g–i) after surgery, new bone tissue could be observed in the pLenti6.3-ctrl group (a, d, g), the pLenti6.3-efnb2siRNA group (b, e, h), and the pLenti6.3-ephb4siRNA group (c, f, i). (j) Statistical analysis of the newly formed bone area among the three groups; nb, new bone; scale bars, 50 *μ*m; ^*∗∗*^ *p* < 0.001 versus pLenti6.3-ctrl group.

New bone tissue could be observed in all of the three groups at 7 days after surgery. In addition, no statistically significant difference in the newly formed bone area was detected among these groups. At 14 days after surgery, the newly formed bone area was significantly lower in the pLenti6.3-ephb4siRNA group than in the pLenti6.3-ctrl group. In contrast, the newly formed bone area was the same in the pLenti6.3-efnb2siRNA group when compared with the pLenti6.3-ctrl group. At 21 days after surgery, bone defects were almost completely repaired by the newly regenerated bone tissue in all groups, and no significant difference was detected among these groups (Figure 6).

**Figure 7:**
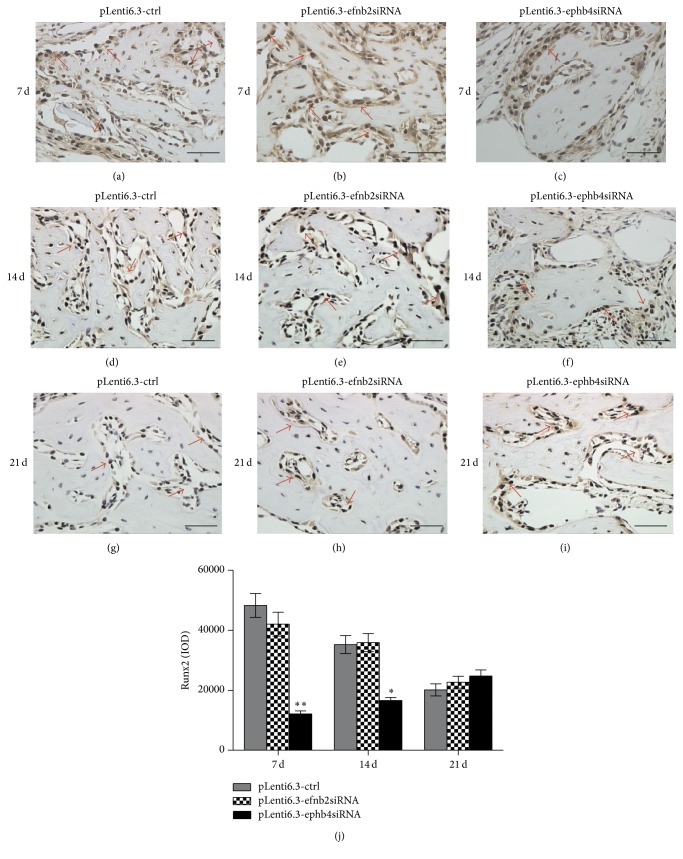
Immunohistochemistry staining for Runx2. (a–i) At 7 days (a–c), 14 days (d–f), and 21 days (g–i) after surgery, different numbers of Runx2-positive cells with brown-stained nuclei could be found surrounding the trabecular bone in the newly formed bone area in the pLenti6.3-ctrl group (a, d, g), the pLenti6.3-efnb2siRNA group (b, e, h), and the pLenti6.3-ephb4siRNA group (c, f, i). (j) Quantitative analysis* via* IOD measurements using the Image-Pro Plus 6.0 software. Arrow, positive Runx2 staining; scale bars, 50 *μ*m; ^*∗*^ *p* < 0.05, ^*∗∗*^ *p* < 0.001 versus pLenti6.3-ctrl group.

**Figure 8:**
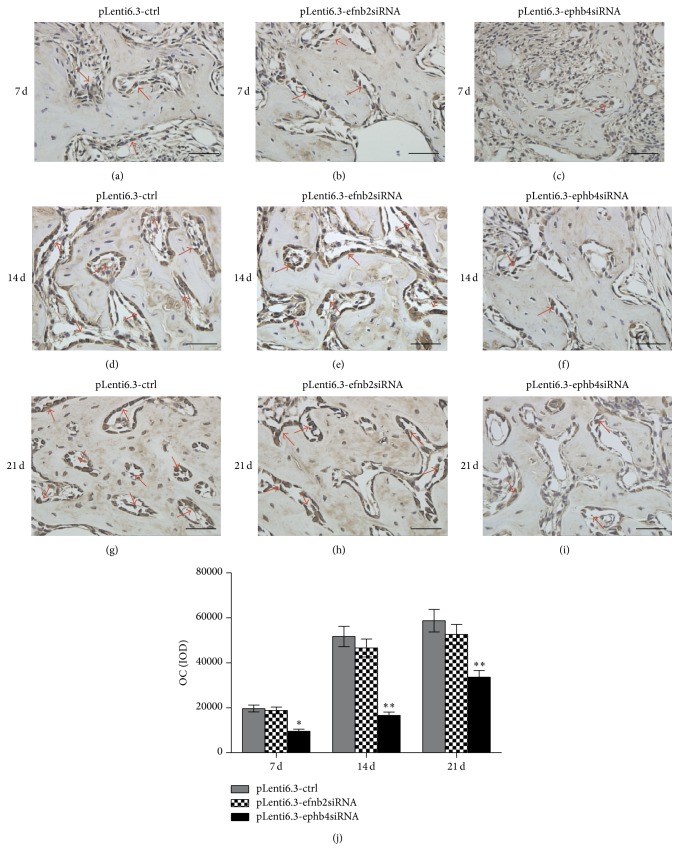
Immunohistochemistry staining for OC. (a–i) At 7 days (a–c), 14 days (d–f), and 21 days (g–i) after surgery, OC-positive staining could be observed in the osteoblasts, osteocytes, and bone matrix in the newly formed bone area in the pLenti6.3-ctrl group (a, d, g), the pLenti6.3-efnb2siRNA group (b, e, h), and the pLenti6.3-ephb4siRNA group (c, f, i). (j) Quantitative analysis* via* IOD measurements using the Image-Pro Plus 6.0 software. Arrow, positive OC staining; scale bars, 50 *μ*m; ^*∗*^ *p* < 0.05, ^*∗∗*^ *p* < 0.001 versus pLenti6.3-ctrl group.

Immunohistochemical staining was also performed to evaluate the in vivo expression of Runx2 and OC proteins. At all time points, we observed stronger Runx2 and OC signals in the pLenti6.3-ctrl group and the pLenti6.3-efnb2siRNA group than in the pLenti6.3-ephb4siRNA group (Figures 7(a)–7(i) and 8(a)–8(i)). Quantitative analysis further confirmed that the IOD values in the pLenti6.3-ephb4siRNA group were significantly lower than those in the pLenti6.3-ctrl group. However, no statistically significant difference in the IOD values was detected between the pLenti6.3-efnb2siRNA group and the pLenti6.3-ctrl group (Figures 7(j) and 8(j)).

### 3.5. Disturbed Expression of EphB4, but Not EphrinB2, Significantly Promoted Osteoclastogenesis in an Inflammatory Microenvironment Created in Mice

**Figure 9:**
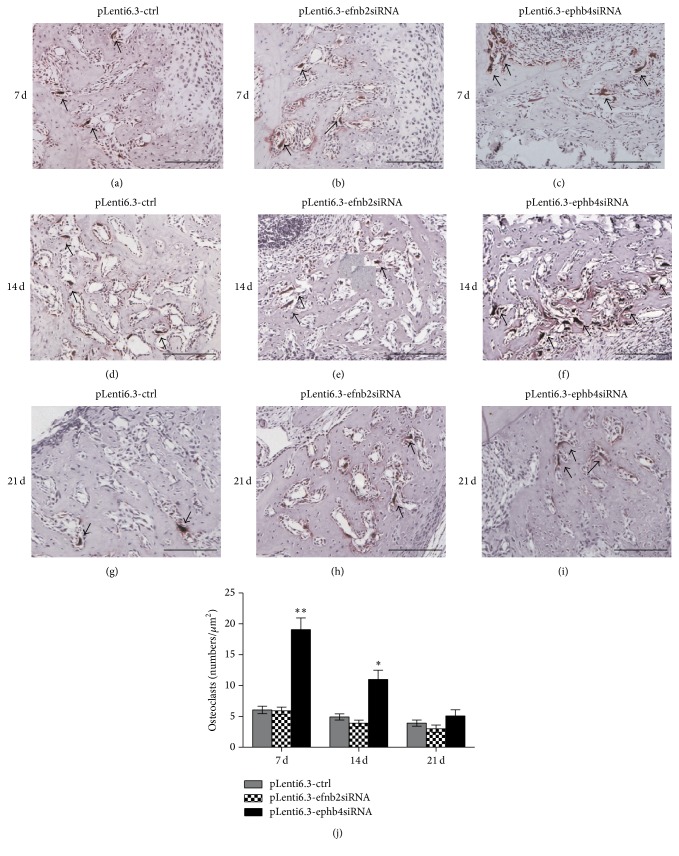
TRACP staining. (a–i) At 7 days (a–c), 14 days (d–f), and 21 days (g–i) after surgery, TRACP staining was performed and the TRACP-positive cells with three or more nuclei were identified as mature osteoclasts in the pLenti6.3-ctrl group (a, d, g), the pLenti6.3-efnb2siRNA group (b, e, h), and the pLenti6.3-ephb4siRNA group (c, f, i). (j) The number of TRACP+ cells with three or more nuclei was counted for quantitative analysis. Arrow, TRACP+ cells with three or more nuclei; scale bars, 100 *μ*m; ^*∗*^ *p* < 0.05, ^*∗∗*^ *p* < 0.001 versus pLenti6.3-ctrl group.

To investigate whether the deficiency of EphB4 or ephrinB2 affected bone resorption during bone regeneration processes in an inflammatory microenvironment, TRACP staining was performed and the TRACP-positive cells with three or more nuclei were identified as mature osteoclasts (Figures 9(a)–9(i)). Quantitative analysis showed a significantly increased number of TRACP+ osteoclasts in the bone defect region in the pLenti6.3-ephb4siRNA group compared to the pLenti6.3-ctrl group (Figure 9(j)). However, no significant difference in the number of TRACP+ multinuclear osteoclasts was detected between the pLenti6.3-efnb2siRNA group and the pLenti6.3-ctrl group (Figure 9(j)).

## 4. Discussion

It has been fully elucidated that cell-cell interaction mediated by ephrinB2 on osteoclasts and EphB4 on osteoblasts generates bidirectional antiosteoclastogenic and proosteoblastogenic signals which presumably facilitates transition from bone resorption to bone formation in a noninflammatory microenvironment. This makes it extremely promising to find out the final role of ephrinB2-EphB4 signaling in osteogenic differentiation, bone formation, and tissue regeneration, especially in the situation of bone disorders with persistent inflammation. In this study we showed that the EphB4 receptor, but not ephrinB2 ligand, plays a more important role in promoting bone repair in an inflammatory microenvironment.

Previously in our in vitro study, we found that the treatment with TNF-*α* at lower concentrations mainly activates the MAPK family members in osteoblasts, which eventually promotes osteogenic differentiation and enhances expression levels of osteogenic transcription factors and bone marker genes. In contrast, higher concentrations of TNF-*α* result in the activation of NF-*κ*B signaling pathway and inhibit osteogenic differentiation. In addition, long-term treatment with TNF-*α* displays dose-dependent inhibition in osteogenic differentiation [15]. Therefore in this study, we first injected TNF-*α* intraperitoneally into male C57BL/6 mice at different doses to find an appropriate TNF-*α* treatment for the establishment of an in vivo inflammatory microenvironment. As shown by our findings, the serum TNF-*α* levels in mice receiving injections of TNF-*α* at the dose of 5 *µ*g/kg displayed a quick increase and reached a steady plateau state as early as 7 days after the first injection. Therefore, intraperitoneal injections of TNF-*α* at the dose of 5 *µ*g/kg were chosen for the following experiments.

Previous studies have demonstrated that suppressing EphB4 signaling is able to decrease expression levels of several osteogenic genes involved in the late osteoblast differentiation [16]. In addition, decreased EphB4 level reduces the in vitro mineral-forming capacity of human MSCs [12] and mouse bone cells [17], which was proved to be the result of inhibition in the expression levels of bone matrix genes associated with mineralization. Furthermore, mice overexpressing EphB4 showed increased bone formation parameters, a significant decrease in osteoclastic activity, and a prominent increase in osteoblastic activity [8]. These data strongly demonstrated that EphB4 signaling plays an important role in promoting osteogenic differentiation and bone formation in a noninflammatory physiological condition. However in the dental clinic, the bone regenerative processes often occur in an inflammatory microenvironment, which is commonly seen in patients suffering from periodontal diseases. Thus it is of great clinical significance to further investigate the role of EphB4 signaling in an inflammatory microenvironment. In this study, we found that in an in vivo inflammatory microenvironment decreased expression levels of EphB4 downregulated expressions of bone master genes Runx2 and Osx, as well as other bone-related genes including ALP, OC, and BSP. Simultaneously, the expression level of the osteoclastogenic marker gene NFATc1 was significantly increased. TRACP staining further confirmed that the numbers of TRACP+ multinucleated osteoclasts were significantly increased by the treatment of siRNAs specifically targeting EphB4 in an inflammatory microenvironment. Moreover, histomorphometric analysis revealed that bone regeneration was significantly retarded in mice receiving both intraperitoneal TNF-*α* injections and lentiviral particles encoding siRNAs specifically targeting EphB4. Together with previous findings by other research groups, our results strongly support that EphB4 not only plays an important role in a noninflammatory microenvironment but also actively participates in bone regeneration processes in an inflammatory microenvironment.

The bidirectional antiosteoclastogenic and proosteoblastogenic signals mediated by ephrinB2 on osteoclasts and EphB4 on osteoblasts are considered to be a tight coupling process in a noninflammatory microenvironment [8]. For example, ephrinB2 overexpression enhances osteogenic differentiation of human mesenchymal stem cells and promotes mineralization in a polyethyleneimine-ephrinB2 gene-activated matrix [18]. In addition, suppressing ephrinB2 signaling inhibits mineralization and downregulates expression levels of several bone-related genes in osteoblasts [16, 19], human MSCs [12], and mouse bone cells [17]. However, in our study, we found that disturbed expressions of ephrinB2 by siRNA treatment showed no effects on bone regeneration in an in vivo inflammatory microenvironment. Although the exact molecular mechanisms underlying the discrepancies still need further investigations, our data suggest that an inflammatory microenvironment may evoke some compensatory signaling pathways to replace the downregulated ephrinB2 ligand. For example, previous reports have indicated that ephrinB1 can maintain the function of EphB4 signaling pathway in the absence of ephrinB2 [8].

In conclusion, downregulated expressions of EphB4 by siRNAs significantly inhibited osteogenic activity, enhanced osteoclastogenic differentiation, and retarded bone regeneration in an in vivo inflammatory microenvironment established by intraperitoneal TNF-*α* injections. However, selected inhibition of ephrinB2 by siRNAs showed no obvious effect on bone remodeling and bone regeneration in the same in vivo inflammatory microenvironment, suggesting that an inflammatory microenvironment may evoke some compensatory signaling pathways to replace the missing functions of ephrinB2 ligand. The in vivo effect of an inflammatory microenvironment on ephrinB2/EphB4 signaling pathway needs further investigations.

## Figures and Tables

**Table 1:** Sequences of the primers for real-time PCR.

Primer	Sequences	
Runx2	Forward:	5′-AGGTCGGTGTGAACGGATTTG-3′
	Reverse:	5′-TGTAGACCATGTAGTTGAGGTCA-3′
OC	Forward:	5′-GCGCTCTGTCTCTCTGACCT-3′
	Reverse:	5′-GCCGGAGTCTGTTCACTACC-3′
Osx	Forward:	5′-ATGGCGTCCTCTCTGCTTG-3′
	Reverse:	5′-TGAAAGGTCAGCGTATGGCTT-3′
BSP	Forward:	5′-CAGGGAGGCAGTGACTCTTC-3′
	Reverse:	5′-AGTGTGGAAAGTGTGGCGTT-3′
ALP	Forward:	5′-CTTGCTGGTGGAAGGAGGCAGG-3′
	Reverse:	5′-GGAGCACAGGAAGTTGGGAC-3′
NFATc1	Forward:	5′-CAAGTCTCACCACAGGGCTCACTA-3′
	Reverse:	5′-TCAGCCGTCCCAATGAACAG-3′
EphrinB2	Forward:	5′-ACGGTCCAACAAGACGTCCA-3′
	Reverse:	5′-GCTGTTGCCATCGGTGCTA-3′
EphB4	Forward:	5′-AGTGGCTTCGAGCCATCAAGA-3′
	Reverse:	5′-CTCCTGGCTTAGCTTGGGACTTC-3′
GAPDH	Forward:	5′-AGGTCGGTGTGAACGGATTTG-3′
	Reverse:	5′-TGTAGACCATGTAGTTGAGGTCA-3′
